# Supplementary material for: Gypenoside XVII Reduces Synaptic Glutamate Release and Protects against Excitotoxic Injury in Rats
Source: Biomolecules. 2024 May 16;14(5):589. doi: 10.3390/biom14050589 (PMC11118014; doi:10.3390/biom14050589)

GP-17_ PKA

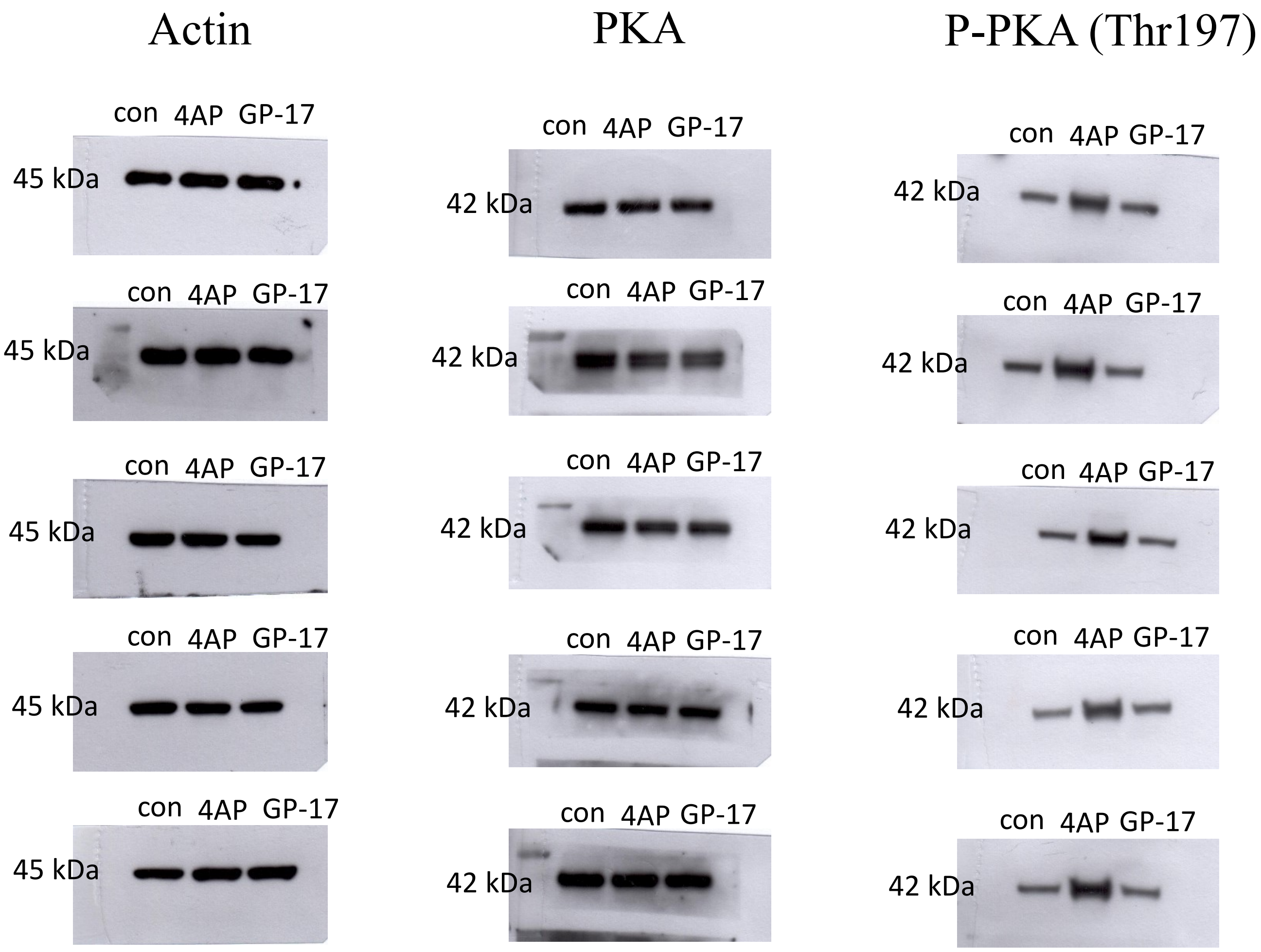

GP-17_ Synapsin I

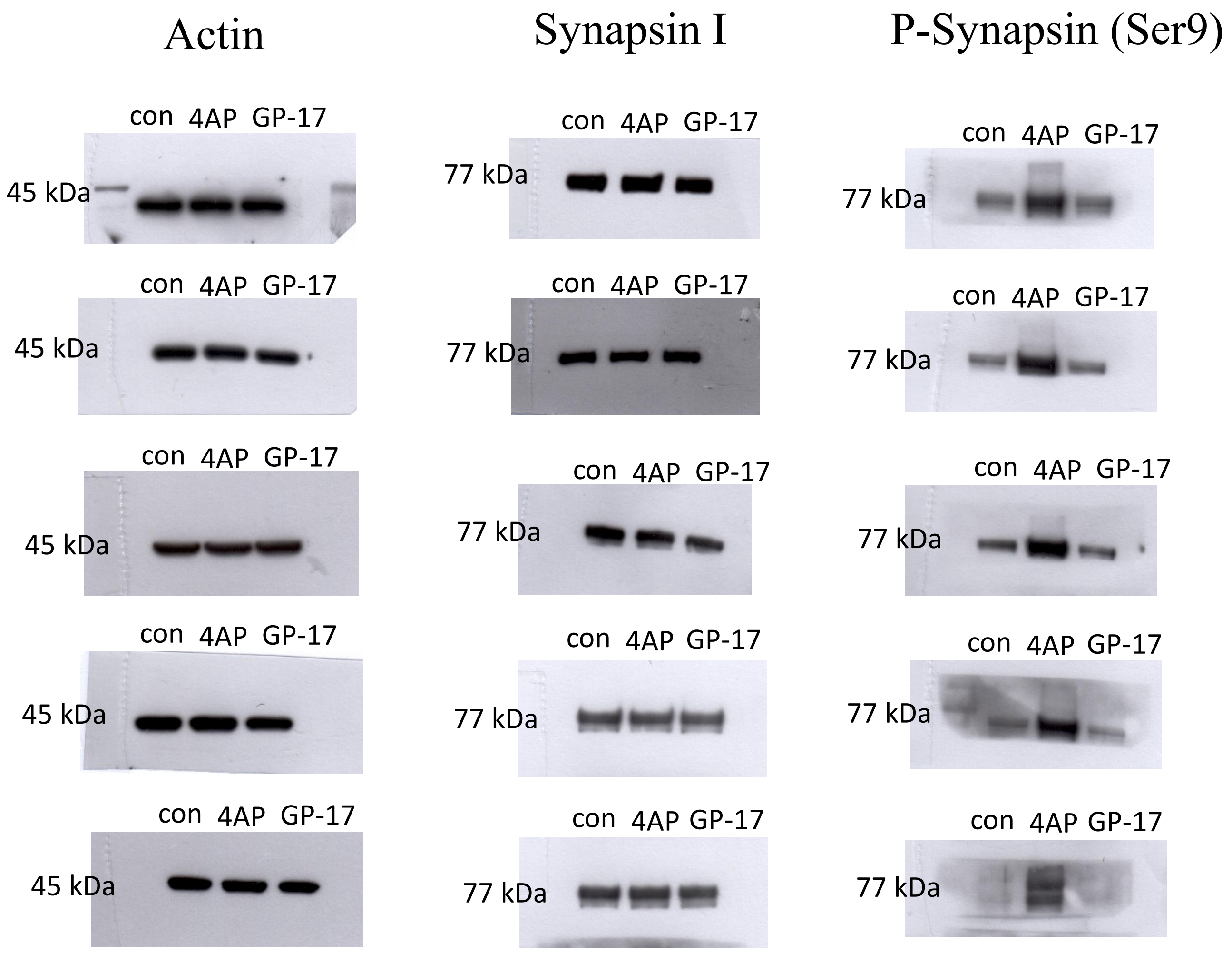

GP-17_ SNAP25

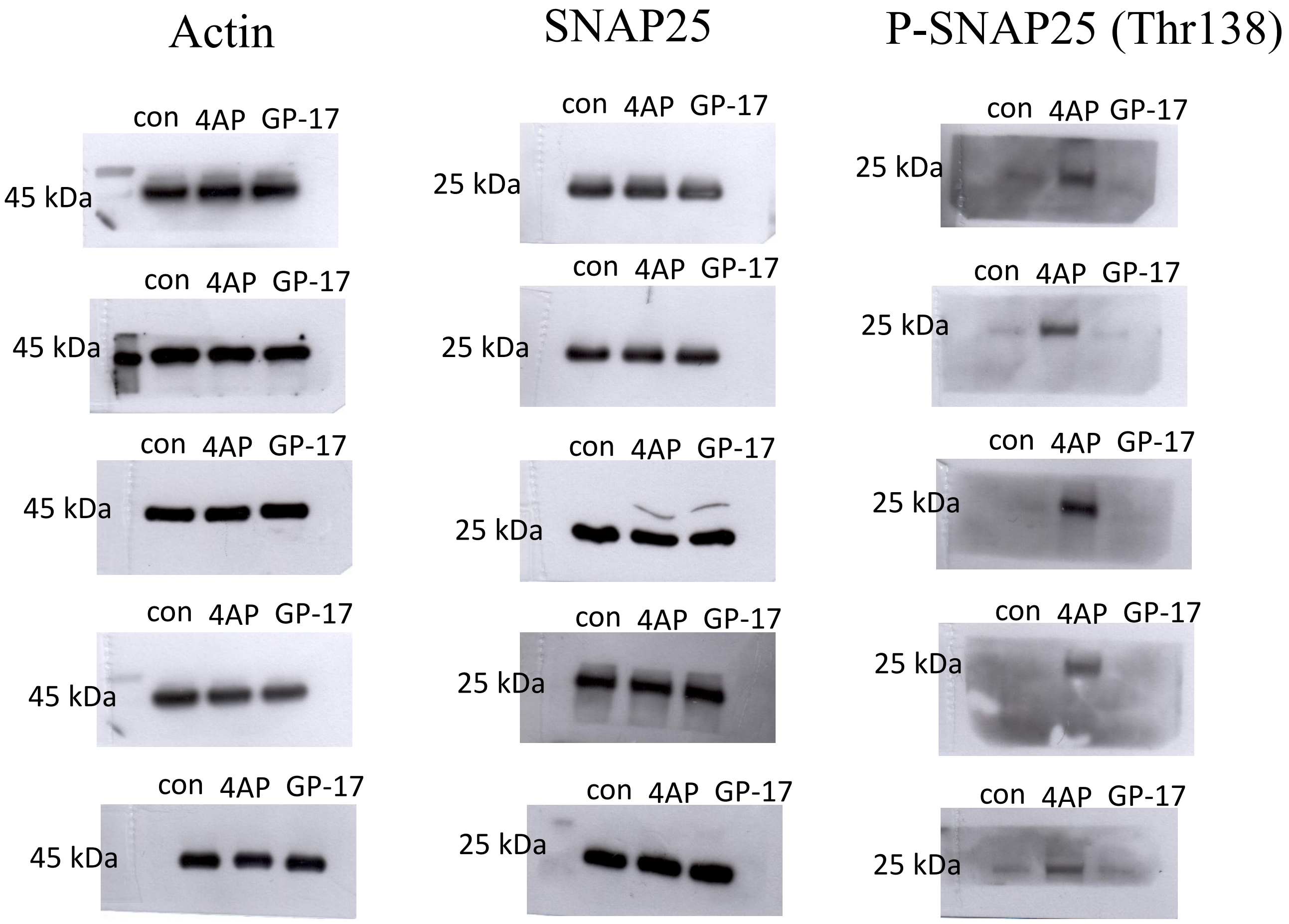

GP-17_Neuron Glutamate transporters

Actin

SNAT1

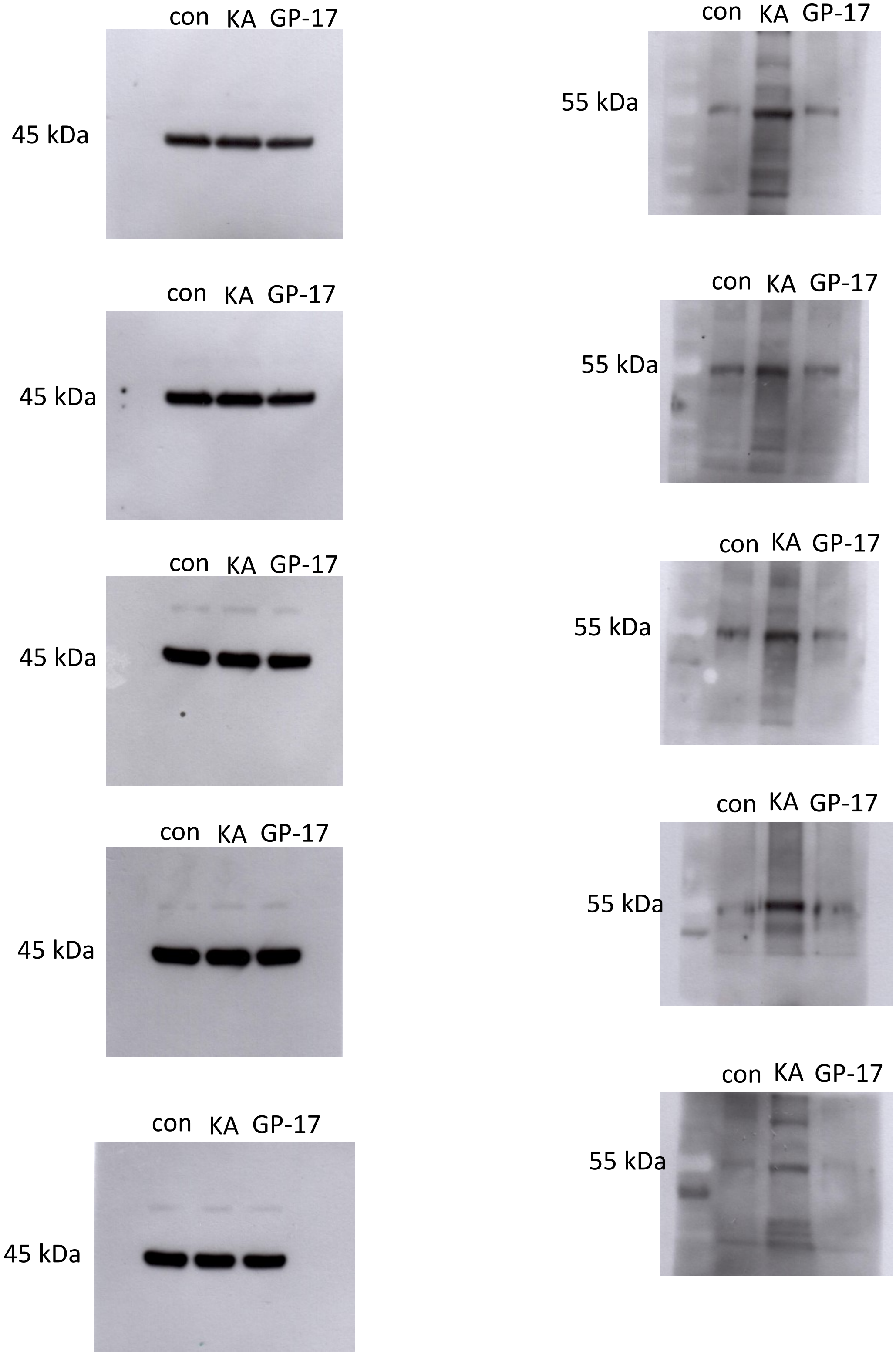

GP-17_Neuron Glutamate transporters

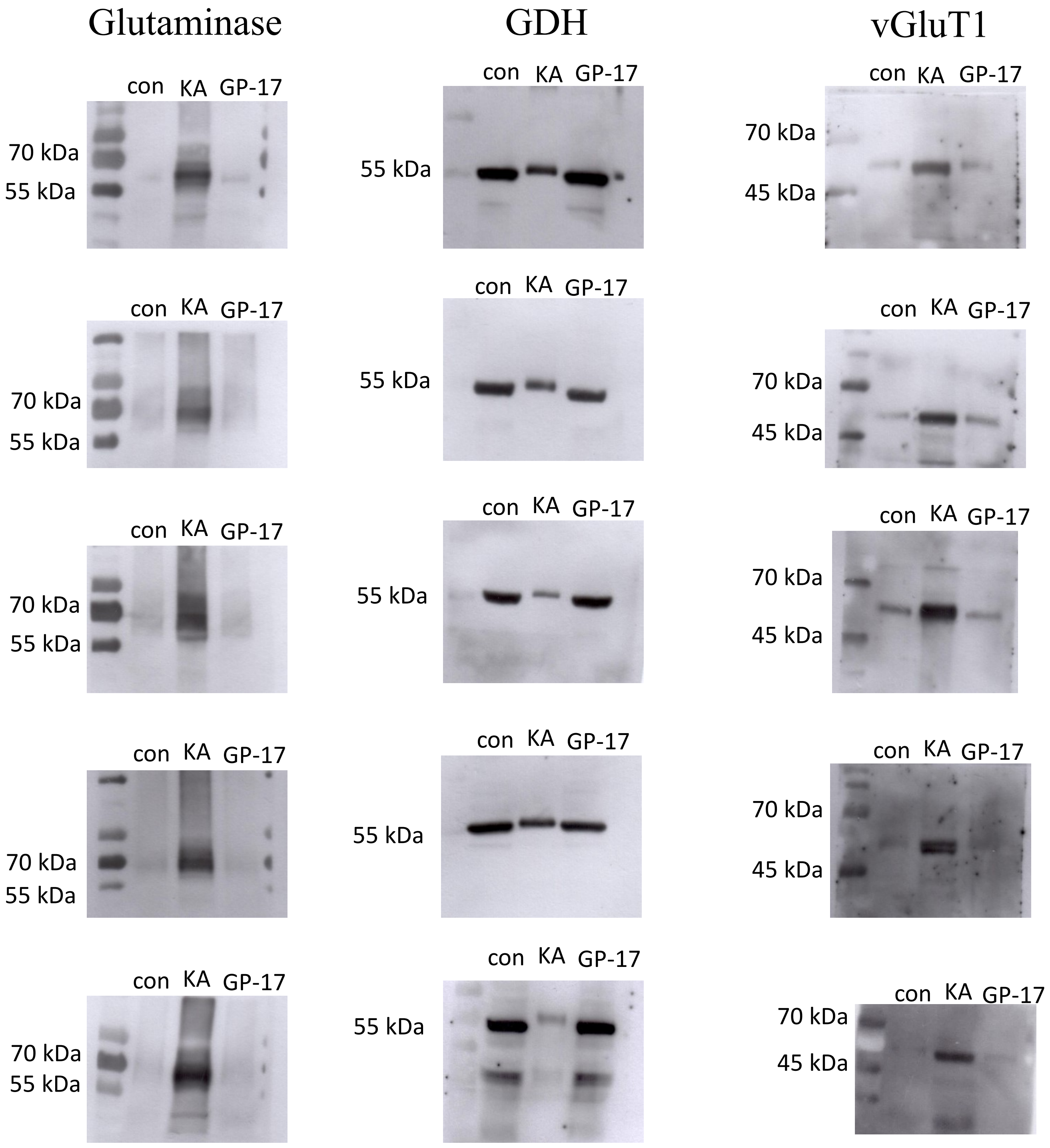

GP-17_NMDA receptor

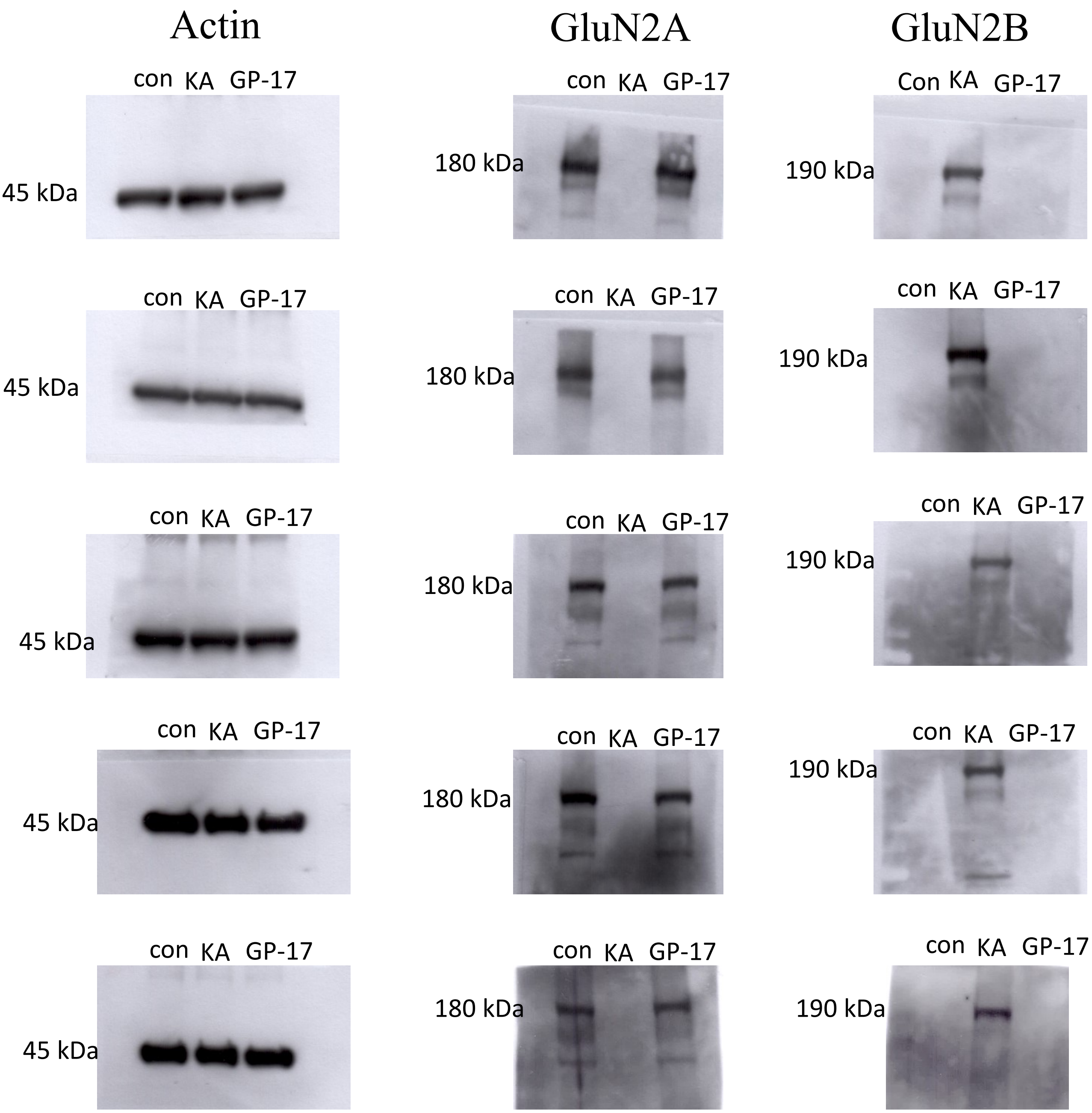

GP-17_ ArgII

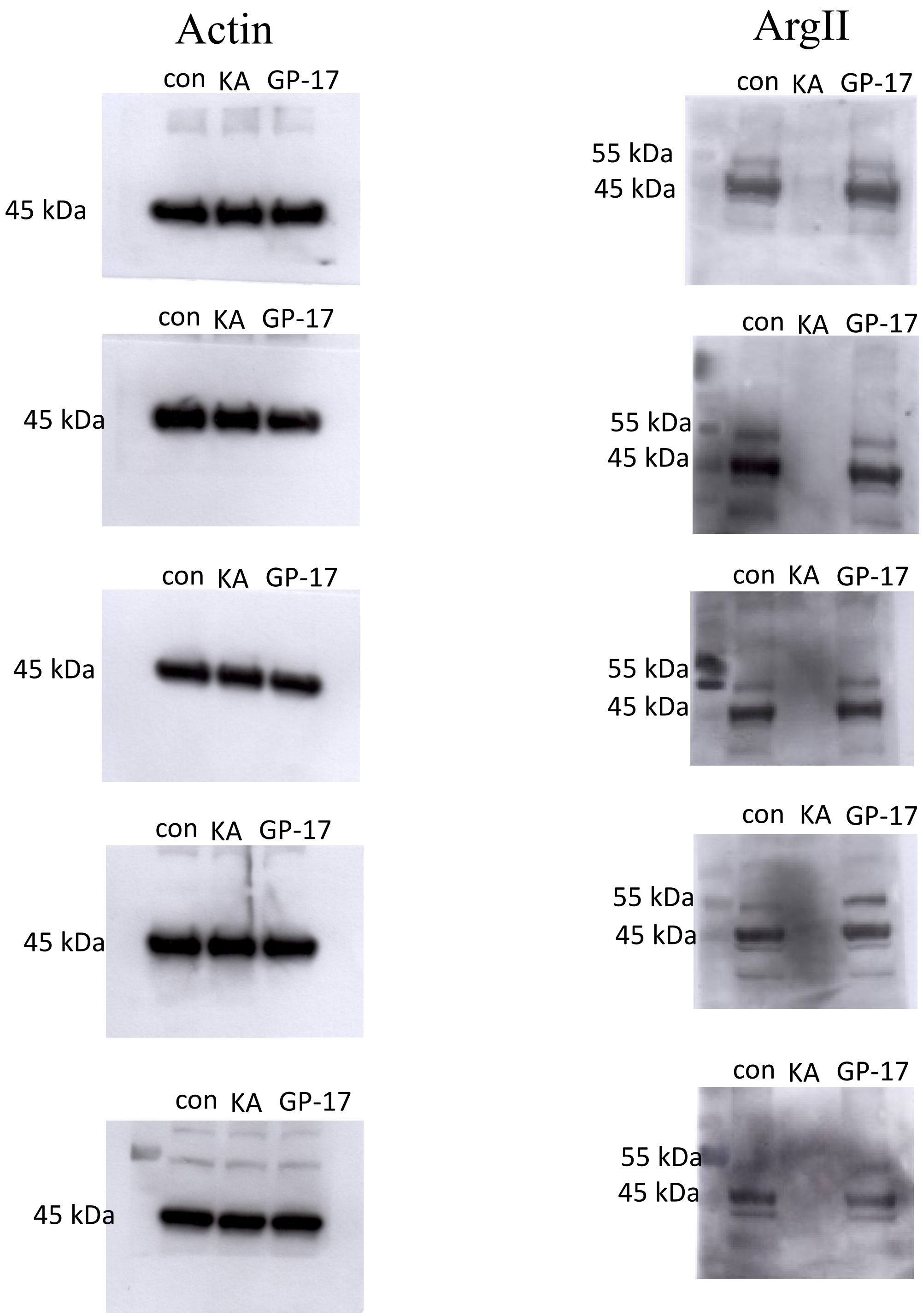
Actin
ArgII
con KA GP-17
45 kDa
55 kDa
45 kDa
con KA GP-17
45 kDa
55 kDa
45 kDa
con KA GP-17
45 kDa
55 kDa
45 kDa
con KA GP-17
45 kDa
55 kDa
45 kDa
con KA GP-17
45 kDa
55 kDa
45 kDa

GP-17_ GluN1

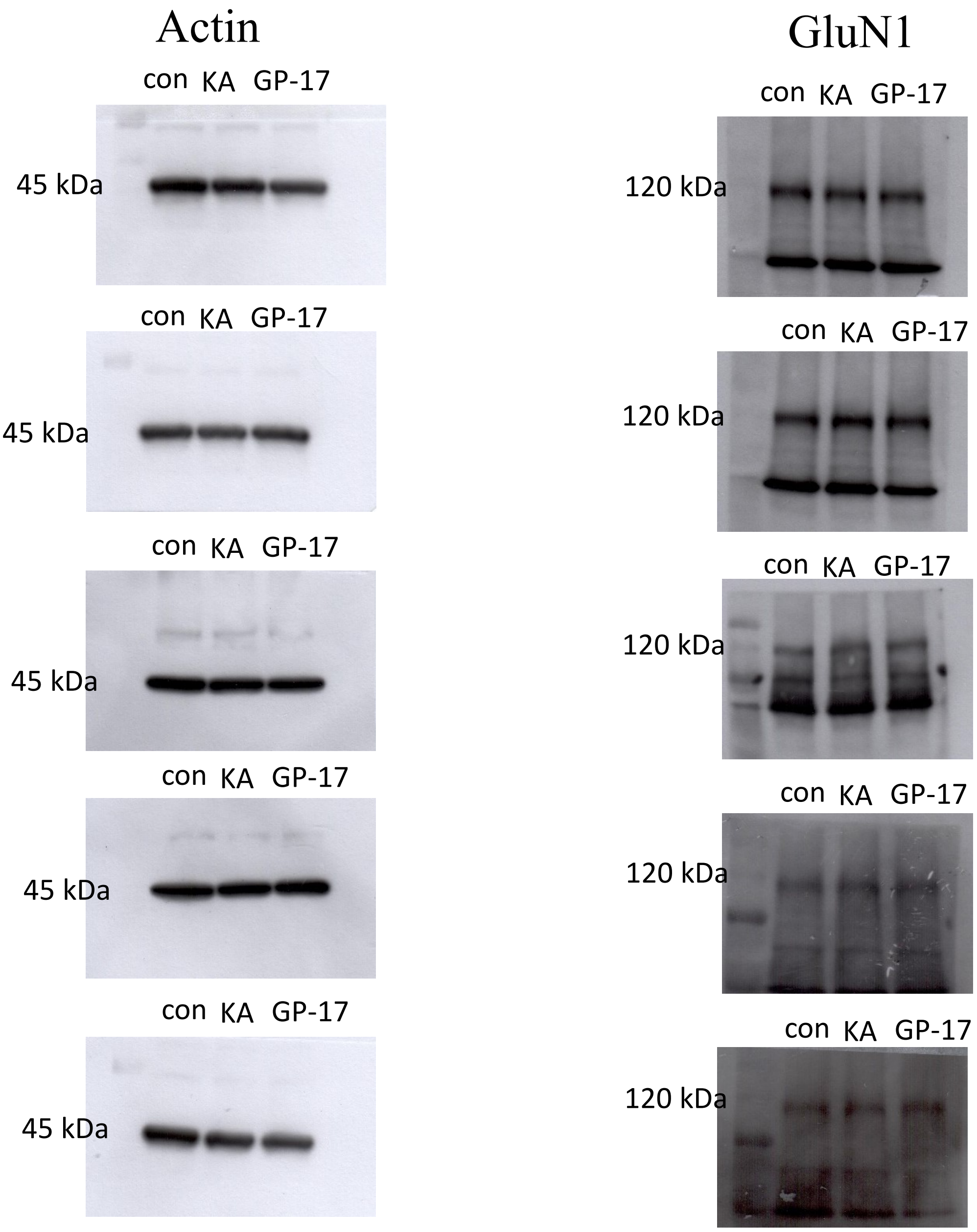

Supplement: Supplementary file 1 [file biomolecules-14-00589-s001.zip › biomolecules-2939571-supplementary.pdf]
